# Recovery of Valuable Materials Based on Pb and Zn in the Hydrometallurgical Processing of Copper Shaft Furnace Dust

**DOI:** 10.3390/ma18091935

**Published:** 2025-04-24

**Authors:** Martina Laubertová, Martin Sisol, Jaroslav Briančin, Jarmila Trpčevská, Michaela Ružičková

**Affiliations:** 1 Institute of Earth Resources, Faculty of Mining, Ecology, Process Control and Geotechnologies, Technical University of Košice, Letná 1/9, 042 00 Košice, Slovakia; martin.sisol@tuke.sk; 2 Institute of Geotechnics SAS (Slovak Academy of Sciences), Watsonová 45, 040 01 Košice, Slovakia; briancin@saske.sk; 3 Institute of Recycling and Environmental Technologies, Faculty of Materials, Metallurgy and Recycling, Technical University of Košice, Letná 1/9, 042 00 Košice, Slovakia; jarmila.trpcevska@tuke.sk (J.T.); michaela.ruzickova@tuke.sk (M.R.)

**Keywords:** hydrometallurgy, copper dust, metals, lead sulfate, zinc oxide

## Abstract

Copper shaft furnace (CSF) dust containing valuable metals with a composition of 44.02% Zn and 14.57% Pb, in the form of oxides (PbO and ZnO), was used for leaching in 1 mol/L sodium hydroxide lixiviant at a temperature of 80 °C. The leaching efficiency for lead removal was 98%. The leaching of CSF dust in sodium hydroxide was thermodynamically studied using Pourbaix diagrams for the Pb/Zn/-Na–H_2_O system at temperatures of 25 °C and 80 °C. A suitable precipitating agent was 0.5 mol/L sulfuric acid at pH 3. The formation of lead sulfate as the final product was confirmed by SEM, EDX, and XRD analysis. Although increasing the temperature reduced the aging time required for the precipitation, it did not affect the amount of lead precipitated. The solution, after lead precipitation and containing zinc (Zn^2+^), was further treated with ammonium carbonate for zinc precipitation. Various analytical methods, including SEM, EDX, XRD, XRF, and AAS, were used to analyze the input samples and the final products obtained after alkali leaching of CSF dust and lead and zinc precipitation.

## 1. Introduction

In conventional primary raw materials, the content of zinc, tin, and lead are varied between 1 and 10% Zn, 3 and 8% Pb, and 0.5 and 4% Sn. Fine-grained industrial wastes such as steel and copper production flue dust can be classified as unconventional resources of strategic materials containing recoverable metals. Dust, which is classified as hazardous waste, is also valuable metal-bearing waste that can be processed using suitable hydrometallurgical methods [1,2,3]. A specific feature of the dust is its high content of non-ferrous heavy metals Pb (14–20%), Sn (0.4–30%), and Zn (32–52%), also As and Cu, respectively, in oxide form [4]. Further processing of these materials to obtain usable products minimizes the environmental impact on the environment and reduces financial costs associated with their storage. The content of heavy metals in dust from secondary copper production varies depending on the furnace equipment used in the production process and is therefore defined as copper shaft furnace (CSF) dust, copper converter (CC) dust, and copper anode (CA) dust [5,6,7,8].

Dust is considered an alternative raw material, and it is necessary to find environmentally acceptable methods for its processing [4]. The recycling potential of dust is based on its hydrometallurgical processing and subsequent recovery of usable products based on Pb, Zn, and Sn, as environmental aspects in hydrometallurgical processing are less significant than in pyrometallurgical production. In EAF dust, the problem is Fe in the form of franklinite (ZnFe_2_O_4_), and in copper flue dust, it is Pb and As [9,10].

The hydrometallurgical methods used to recover metals from solutions after leaching include precipitation, crystallization, reduction of metals from solutions using gases, cementation, electrolysis, ion exchange, or solvent extraction [11,12].

Hydrometallurgical processing of industrial metal-bearing waste containing Zn, Pb, and Sn (dust, slag, sludge, and solid residues) has been developed by several authors worldwide. Dust can be leached in alkaline and acidic leaching media to obtain/remove lead or arsenic and recover zinc using conventional as well as intensification methods. By these methods, 88 to 99.6% of Pb was leached into ionic form in the solution [4,10,13,14,15].

Methods such as precipitation or cementation can be used to extract lead from the solution. The solution, cleaned of lead and other impurities, is subjected to further processing to obtain a usable product based on Zn or Pb, depending on the leaching medium used. Table 1 provides a detailed overview of the leaching media and conditions used in processing different types of dust and solid residues after leaching.

**Table 1:** Leaching medium and conditions used for leaching materials based on Pb and Zn.

Reference	Material *	Qualitative Analysis	Quantitative Analysis	Leaching	
				Medium	Conditions
[16]	EAF dust	ZnO, ZnFe_2_O_4_, PbO, Fe_2_O_3_.	7–40% Zn, 4–9% Pb, 24–27% Fe.	10 M NaOH.	T = 95.8 °C, t = 120 min, L/S = 7:0.1.
[17]	SZL residue	PbSO_4_ PbO, Fe_2_O_3_, ZnFe_2_O_4_.	7.98% Zn, 19.02% Pb.	11% NaOH.	p = 1 bar, T = 100 °C, t = 60 min, 700 rpm.
[18]	EAF dust	ZnFe_2_O_4_, Fe_3_O_4_, ZnO, PbO.	33.16%, Zn, 1.64% Pb.	0.8 M Citric acid and O_2_.	T = 40 °C, t = 60 min, 500 rpm, O_2_ = 2000 mL/min.
[5]	CA dust	ZnO, Zn_2_SnO_4_, SnO_2_, PbCl_2_, SnCl_2_.	28.35% Zn, 10.28% Pb, 0.67% Fe, 1.5% Sn.	1 M H_2_SO_4_.	L/S = 10, T = 25 °C, t = 10 min, 300 rpm.
[9]	CC dust	ZnO, PbO, SnO_2_.	9.3% Pb, 29.90% Zn, 0.52% Fe.	Acetic acid.	T = 25 °C, 400 rpm, L/S = 40, t = 60 min.
[19]	BF dust	ZnO, ZnSO_4_, ZnS, ZnFe_2_O_4_, Fe_2_O_4_, Fe_3_O_4_	36.16% Fe, 51.27% Fe_2_O_3_, 11.84% Zn.	3 M NH_4_Cl.	t = 90 min, T = 70 °C, L/S = 10 mL/g, A = 400 rpm.
[20]	LSF dust	ZnO, PbSO_4_. Pb_4_(SO_4_)(CO_3_)_2_(OH)_2_.	44.27% Zn, 27.92% Pb.	7 M NH_4_Cl.	T = 100 °C, L/S 10 mL/g, 450 rpm, t = 60 min.
[21]	SZL residue	PbSO_4_, SnO_2_.	0,46% Sn, 54% Pb, 4.28% Zn.	0.5 M H_2_SO_4_ 24 g/L oxalic acid.	T = 60 °C, t = 30 min, L/S 10 mL/g, 400 rpm.
[22]	CCM dust	PbSO_4_, ZnSO_4_.H_2_O.	5.91%, 3.04% Cu, 25.06% Pb, 13% As.	H_2_SO_4_.	S:L = 1.5; t = 60 °C; τ = 60 min, pH = 0.8–1.0, 300 rpm.

* EAF—electric arc furnace; BF—blast furnace; CA—copper anode; CC—copper converter; LSF—lead slag fuming; SZL—solid zinc leaching; CCM—copper converter matte.

Copper shaft furnace dust, studied in this research, characterized by a high content of Pb (14–20%) and Zn (30–49%), is produced from secondary copper production. Lead and zinc in shaft dust are mainly present in the form of PbO and ZnO oxides [4].

The objective of this study is to investigate the direct use of alkaline leaching for processing CSF dust containing lead and zinc. The conditions for the chemical precipitation of lead from the solution at the ambient temperature were examined. The resulting precipitate, lead sulfate, is the final product, and zinc remains in the solid residue for further processing. Part of the zinc (Zn^2+^) that passes into the solution can be further processed. The novelty of this study is the direct application of alkaline leaching of copper dust for Pb recovery and selective precipitation of PbSO_4_ as a final marketable product under the investigated conditions. This results in the valorization of industrial hazardous waste with the aim of obtaining usable products and reducing the environmental impact.

## 2. Materials and Methods

### 2.1. Experimental Samples and Reagents

The CSF dust representative samples used in this study were collected by multiple quartering and homogenization from a Slovak copper recycling company. The bulk dust samples from the smelter were collected over ten days, and sample preparation was performed using cone, quartering, and split methods to obtain a representative sample of CSF dust. The grain size of the sample was less than 1 μm of the small particle fraction. This particle size of the CSF dust sample is sufficient for chemical analysis without further treatment. After drying, five analytical samples were taken from this representative sample for chemical analysis using the atomic absorption spectrometry (AAS) method. The leaching medium in this study included sodium hydroxide (NaOH). Sulfuric acid (H_2_SO_4_) was used as the pH regulator. The usage, purpose, and properties of the leaching reagents are detailed in Table 2.

### 2.2. Analytical and Experimental Methods

For a representative sample, a CSF dust hot air dryer (HS12A/198, Chirana Brno, Brno, Czech Republic) was used. 

EDX-7000P spectrometer (Schimadzu, Kyoto, Japan) X-ray fluorescence spectrometer (XRF) was used for indicative multi-element analysis of CSF dust and precipitation samples. 

Chemical analysis of copper, nickel, zinc, lead, and iron in the samples was performed using atomic absorption spectroscopy (AAS) on a Varian spectrophotometer AA20+ with a detection limit of 0.3–6 ppb (Varian, Belrose, Australia). The most suitable working conditions for the efficiency of AAS measurements of fuel flow, acetylene, and burner height were experimentally determined before the analyses using a calibration solution with the highest content of a particular element. Each leachate solution was measured three times to ensure accuracy.

Phase analysis was performed using an X-ray powder diffractometer (Philips X’Pert PRO MRD) with Co-Kα radiation and a measurement range of 10–120° 2θ, with a scan step of 0.0170° (Philips, Amsterdam, The Netherlands). The samples were prepared according to the standardized Panalytical backloading system, which provides an almost random distribution of particles.

A scanning electron microscope MIRA3 FE-SEM (TESCAN, Prague, Czech Republic) was used to observe the morphology and determine the particle sizes; SEM also enabled a semi-quantitative elemental analysis via EDX. EDX data were processed using AZtec software v6.0 (Oxford Instruments, Oxford, UK). The redox potential (E) and pH were measured using an Orion Lab Star PH111 pH meter (Thermo Fisher Scientific, Waltham, MA, USA).

Pourbaix Eh–pH diagrams were employed to evaluate the leaching thermodynamics using HSC Chemistry, version 10.0.2.3, with a University Basic license (Metso Finland Oy, Espoo, Finland). Predicted speciation diagrams in aqueous solutions were generated using MEDUSA software (version 32-bit, 2010, Royal Institute of Technology, Stockholm, Sweden) (Make Equilibrium Diagrams Using Sophisticated Algorithms) [23].

## 3. Results and Discussion

Hydrometallurgical procedures were carried out using a standard leaching apparatus under atmospheric pressure. The initial volume of the leaching reagent was 200 mL of 1 M NaOH in a plastic reactor. A 10 g sample of CSF dust was leached at 80 °C, and the ratio of liquid-to-solid phase (L:S) was 20. Aliquots of 5 mL were taken at different leaching times for the determination of Pb and Zn content in solution using AAS. The average of five analytical samples of CSF dust, with a composition of 44.02% Zn, 14.57% Pb, 1.2% Cu, and 1.59% Sn, along with standard deviation and variance, is shown in Table 3.

**Figure 1:**
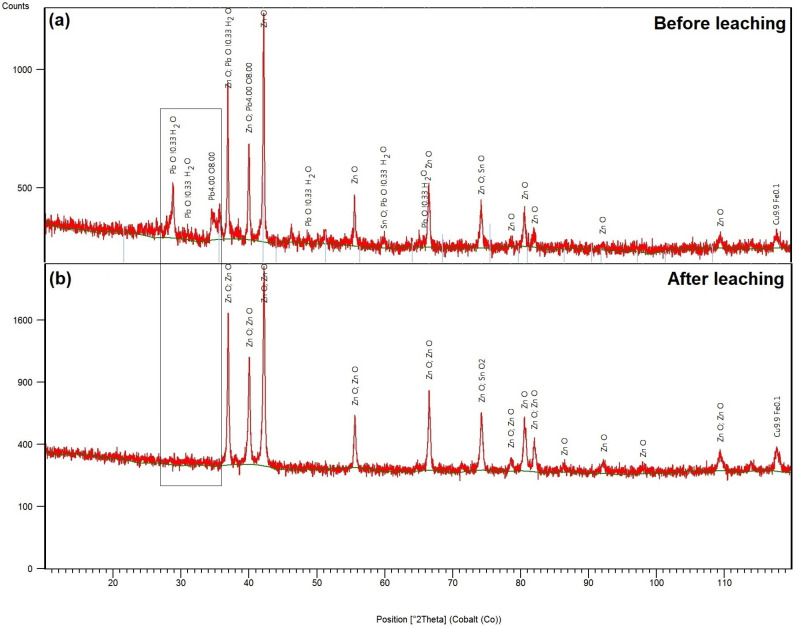
X-ray diffraction (XRD) patterns of CSF dust (**a**) before leaching; (**b**) after leaching.

The qualitative phase diffraction analysis of the CSF dust sample before leaching shows patterns in Figure 1a and was performed using the XRD method. Zn was present in the dust as zinc oxide (ZnO) and Pb as lead oxide (PbO), and hydrated lead oxide (PbO·0.33H_2_O) was identified.

**Figure 2:**
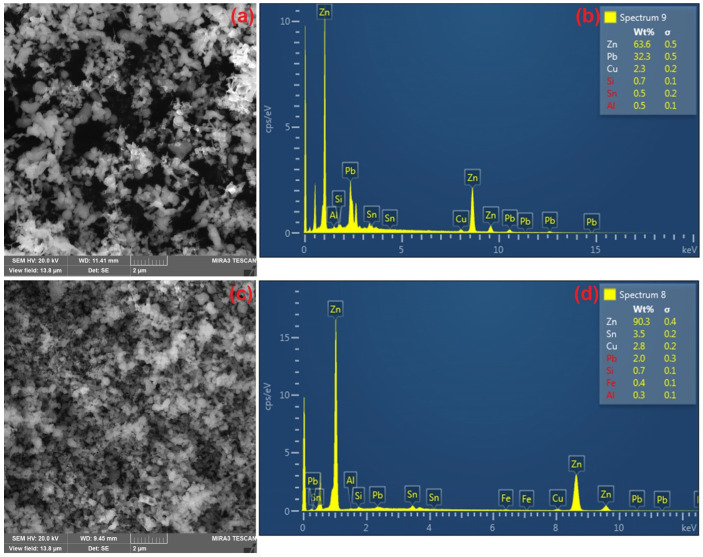
Morphology of CSF dust SEM analysis: (**a**) CSF dust before leaching; (**c**) solid residue after leaching, and EDX analysis: (**b**) CSF dust before leaching; (**d**) solid residue after leaching.

The morphology of the CSF dust sample before leaching was observed via SEM analysis indicating the presence of smaller particles (Figure 2a), while EDX analysis quantified the zinc, lead, and copper content (Figure 2b). Particles have a size smaller than 1 µm and an almost spherical shape.

### 3.1. Thermodynamic Study of Alkaline Leaching of CSF Dust

**Figure 3:**
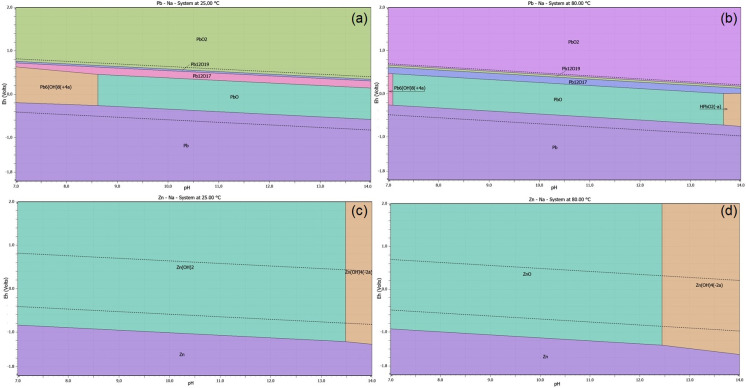
The Pourbaix Eh–pH diagrams of leaching of PbO and ZnO in aqua solution of NaOH at temperatures of 25 °C and 80 °C: (**a**) the Pb–Na–H_2_O system at a temperature of 25 °C; (**b**) the Pb–Na–H_2_O system at a temperature of 80 °C; (**c**) the Zn–Na–H_2_O system at a temperature of 25 °C; (**d**) the Zn–Na–H_2_O system at a temperature of 80 °C;. Note: the dotted lines mark the water stability boundaries.

To determine the conversion of metals (Pb, Zn) from dust, potential-pH diagrams were generated using HSC Chemistry software, version 10.0.2.3 [24]. The phase composition of the CSF dust confirms the presence of Zn and Pb as oxides (ZnO and PbO). An Eh–pH diagram for the element and leaching medium at 25 °C and 80 °C was generated to determine which temperature was more appropriate for leaching, as shown in Figure 3.

Based on the research background, NaOH was selected as the leaching agent for the thermodynamic study of CSF dust leaching. The molality of the major elements was maintained at 1 mol/kg H_2_O, with the pressure kept at 101.325 kPa. The forms of Pb and Zn within the water stability boundaries were determined using Eh–pH diagrams, and a chemical equation was subsequently developed. Thermodynamic analysis using the Van’t Hoff equation indicates that the chemical reactions (1) and (2), shown in Table 4, present a high probability of product formation.

Figure 3a,b show the Eh–pH diagrams for the Pb-Na-H_2_O system at 25 °C and 80 °C, while Figure 3c,d show the Eh–pH diagrams for Zn-Na-H_2_O leaching at 25 °C and 80 °C. Based on the thermodynamic study of these diagrams, CSF dust samples are not expected to dissolve (leach) in NaOH solution at 25 °C. At the initial desulfurization stage, the Eh–pH of the system reached a value of 14.00, and lead hydroxide rapidly dissolved into the solution in the form of $Pb(OH{)}_{4}^{2-}$. At 80 °C, however, a narrow stability region emerges at pH 14, where both Zn and Pb are transferred into the solution under optimal conditions: 1 M NaOH, a liquid-to-solid ratio of 20, and a 10-min leaching time. Thermodynamic analysis confirms that increasing the temperature to 80 °C is necessary for the desired transfer of metals into solution during alkaline leaching. The stability regions of soluble $PbO_{2}^{2-}$ and $Zn(OH{)}_{4}^{2-}$ complexes are extended with increasing temperature. The stability regions for Pb and Zn oxides shift with temperature because lower temperatures decrease the solubility of Pb oxide. The leaching temperature was constant at 80°C during the experimental procedures. More aggressive leaching conditions of CSF dust, such as a higher temperature or acidity, may favor the leaching process. Therefore, the recovery of Pb can be enhanced by adjusting the pH and Eh values.

### 3.2. Thermodynamic Study of Precipitation Pb from Alkaline Solution

**Figure 4:**
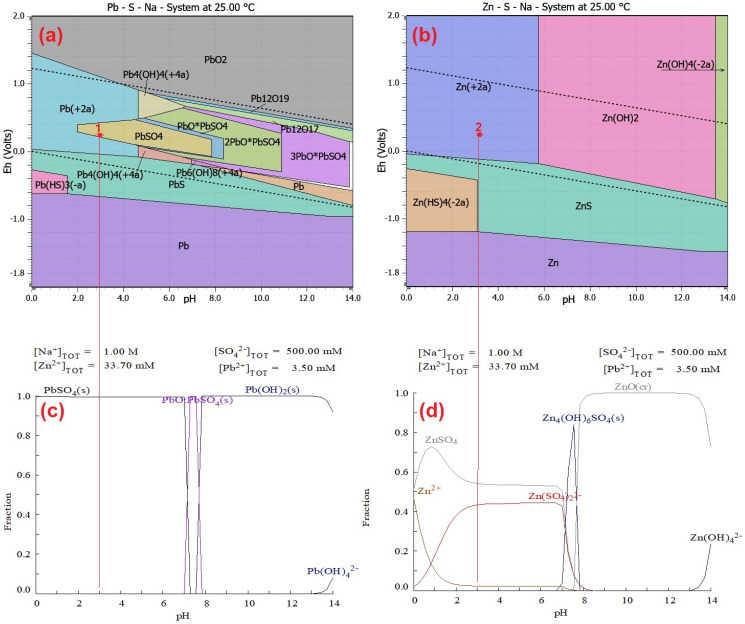
Predicted Pourbaix Eh–pH diagrams for precipitation Pb by H_2_SO_4_ from alkaline leachate (**a**) Pb-S-Na at 25 °C; (**b**) Zn-S-Na at 25 °C; (**c**) predicted fraction diagrams of the PbSO_4_-NaOH-H_2_O system with [Pb^2+^]_TOT_ = 3.50 mM and [$SO_{4}^{2-}$]_TOT_ = 500.00 mM at 25 °C at the Eh of 0.22 V and pH 3; (**d**) ZnSO_4_-NaOH-H_2_O system with [Zn^2+^]_TOT_ = 33.70 mM and [$SO_{4}^{2-}$]_TOT_ = 500.00 mM at 25 °C at the potential Eh = 0.22 V and pH 3.

The alkaline leaching process is followed by the precipitation stage, which aims to recover lead by forming a precipitate based on PbSO_4_ as a final usable product. The thermodynamic conditions for this step are detailed in the reactions (3) and (4) in Table 4. Eh–pH diagrams for various systems were studied to understand the precipitation process. Figure 4a shows the Eh–pH diagram for the Pb-Na-S-H_2_O system for lead precipitation using H_2_SO_4_ after alkaline CSF dust leaching, whereas Figure 4b illustrates the Eh–pH diagram for the Zn-Na-S-H_2_O system. It indicates that at a pH value of 3, zinc remains dissolved in the solution, while lead is removed through the formation of the insoluble compound PbSO_4_ at ambient temperature.

Based on the thermodynamic study of precipitation reactions, a temperature of 25 °C is sufficient for precipitate formation. An important factor in the precipitation process is the stability of the resulting products, where the solubility constant (Ks) of PbSO_4_ is 1.514 × 10^−8^, predicting the formation of a precipitate at a temperature of 25 °C. Figure 4c,d show the fractions of Zn and Pb ions in the sulfate aqueous solution (H_2_SO_4_) at different pH values. The pH for Pb^2^⁺ precipitation via hydrolysis was 3, and the pH in the absence of $SO_{4}^{2-}$ was different. According to the literature [24], as NaOH is consumed, the solution’s pH gradually decreases to about 12.50. During this time, Na_2_[Pb(OH)_4_] continuously precipitated due to the reaction with PbSO_4_. In Figure 4a (red 1), a prediction of lead precipitate in the form of PbSO_4_ is shown, while in Figure 4b (red 2), a prediction of zinc in solution (Zn^2^⁺) is illustrated.

### 3.3. Alkaline Leaching of CSF Dust and Precipitation of Lead

**Figure 5:**
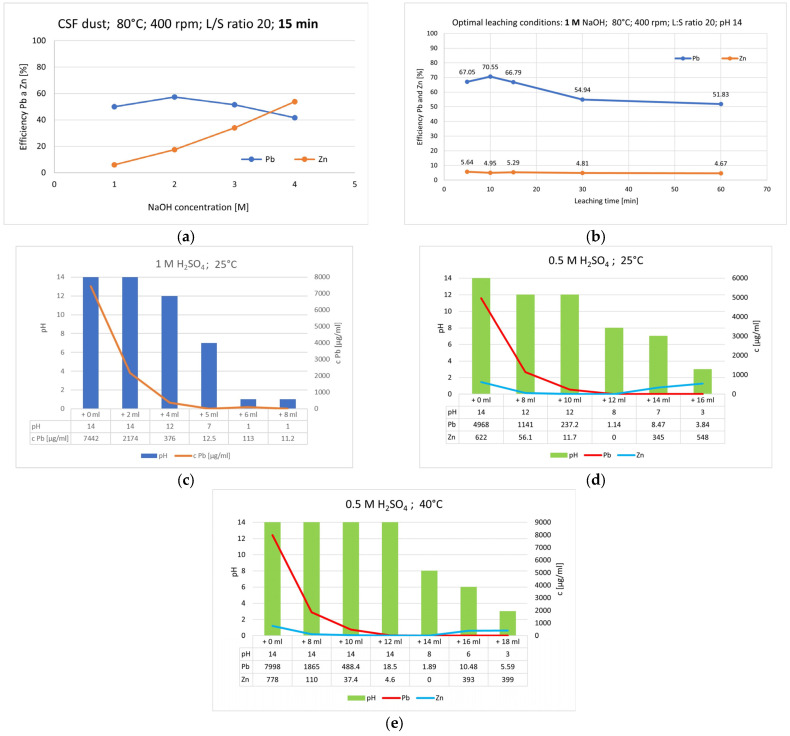
(**a**) Comparing Pb and Zn leaching efficiency at 80 °C in 1, 2, 3, and 4 M NaOH at 15 min. Leaching of CSF dust [25]; (**b**) Pb and Zn leaching efficiency under optimal leaching conditions at 60 min. Leaching of CSF dust in 1 M NaOH at 80 °C; (**c**) precipitation of Pb from solution by 1 M H_2_SO_4_ at 25 °C; (**d**) precipitation of Pb from solution by 0.5 M H_2_SO_4_ at 25 °C; (**e**) at 40 °C.

According to authors [25], the leaching process shown in Figure 5a,b, where different concentrations of NaOH (1, 2, 3, and 4 M NaOH) on the dissolution of Pb and Zn into the solution was monitored in 15 min of leaching. The decrease in Pb recovery with increasing leaching time (Figure 5b) may be due to the reprecipitation of lead.

Based on the obtained results, 1 M NaOH was identified as the more suitable leaching agent, as it achieved higher lead recovery at a temperature of 80 °C. Increased NaOH concentrations promoted zinc recovery. Zinc recovery remained nearly constant, while lead recovery peaked after 10 min of leaching and gradually decreased with prolonged leaching duration.

The XRD phase analysis pattern of the solid residue after leaching is presented in Figure 1b. The diffractogram shows the remaining ZnO phase. Lead oxides are no longer detectable in the sample, confirming their near-complete transfer into the solution or significant removal during leaching. Figure 2c,d illustrate the morphology of the solid residue after leaching, observed using SEM, and EDX analysis, revealing smaller particle sizes compared to the initial sample. It was observed that leaching led to partial particle refinement, with a noticeable reduction in particle size. SEM analysis of the precipitate indicated the presence of fine particles and confirmed the successful precipitation of lead. Additionally, EDX analysis confirmed a change in chemical composition, with lead being either completely removed or significantly reduced.

EDX analysis of the post-leaching CSF dust sample detected the presence of zinc, copper, and tin. A solution of H_2_SO_4_ (0.5 M and 1 M concentrations) was used as a precipitating agent to remove lead from the leachate solution at temperatures of 25 and 40 °C (Figure 5c–e). After each incremental addition of the precipitating agent (8 mL, 10 mL, 12 mL, etc.), the precipitate was left to age, and then the solution was filtered, and the pH was measured. A sparingly soluble compound is precipitated until equilibrium is reached between the precipitated salt and its ions in the saturated solution. The chemical potential at equilibrium equals the sum of the chemical potentials of the ions in the solution, within the range of 0.0 V to 1.1 V.

**Figure 6:**
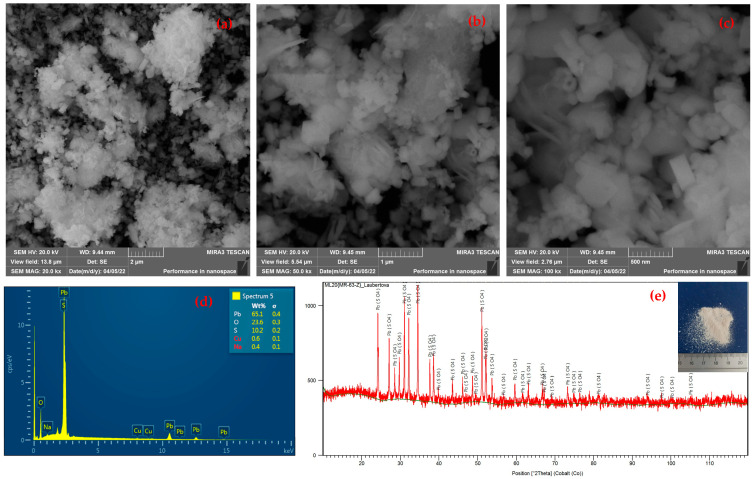
SEM analysis of precipitate PbSO_4_: (**a**–**c**) different magnifications; (**d**) EDX analysis precipitate; (**e**) XRD patterns of precipitate.

The filtrate was analyzed to determine the residual lead content. With each addition of the precipitating agent a 1 M NaOH solution, the Pb content in the solution decreased as it was removed in the form of a precipitate at elevated temperatures and under conditions of pH = 3.1 and Eh = 0.22 V. Under these conditions, zinc was also co-precipitated. At ambient temperature and pH 3, almost all lead precipitated (0.08% residual Pb in solution), while zinc remained mostly dissolved (88.1% Zn in solution). Morphological changes in the precipitates were not observed with variations in higher temperatures. Figure 6a–c show the morphology of the precipitate formed during the precipitation process, where clusters of individual particles can be observed by SEM analysis. EDX analysis of the precipitate confirmed the presence of lead, oxygen, and sulfur (Figure 6d). XRD analysis definitively identified the white precipitate as PbSO_4_ (Figure 6e).

## 4. Conclusions

The current study focuses on the hydrometallurgical processing of CSF dust and investigates the selective removal of lead from the solution after alkaline leaching. This process results in a usable lead sulfate anglesite (PbSO_4_) product in solid form, while zinc remains in the solution for further recovery. The solid residue can be used in a second leaching stage in an acidic medium using sulfuric acid (H_2_SO_4_) or 4 M NaOH to recover zinc into the solution. In addition, the following conclusions were drawn from the experimental study:

- The optimal conditions for the leaching of CSF dust were 1 M NaOH at 80 °C, a liquid-to-solid ratio of 20, and a leaching time of 10 min, resulting in 70.55% lead recovery efficiency.
- It was confirmed that a suitable precipitating agent is 0.5 M H_2_SO_4_ at pH 3.1 and Eh 0.22 V at 25 °C. Although increasing the temperature reduced the time required for the precipitate to age, it did not affect the amount of lead precipitated from the alkaline solution.
- The newly recovered material (PbSO_4_), produced from industrial hazardous waste as an unconventional raw material, can be used as a by-product in lead-acid battery paste production.

## Figures and Tables

**Table 2:** The usage, purpose, and properties of the leaching reagents.

No	Name	Content	Purity	Usage	Purpose	Producer	City/State
1	NaOH	98 (%)	Analytical	1 M aq. solution	Leaching medium	Centralchem	Bratislava/Slovakia
2	H_2_SO_4_	96 (%)	Analytical	0.5 M aq. solution	Precipitant	Penta Chemicals Unlimited	Prague/Czech Republic

**Table 3:** Chemical analysis of main metallic elements in the CSF dust by AAS method.

Content [wt. %]	Zn	Pb	Sn	Fe	Cu	Ni	Cl^−^	L.O.I. ^1^
Average	44.02	14.57	1.59	0.232	1.2	0.02	30.58	7.788
Standard Deviation	5.642	1.791	0.04	0.021	0.081	4 × 10^−3^	3.51	-
Variance	31.83	3.207	0.002	0.021	0.007	2 × 10^−3^	12.37	-

^1^ L.O.I., loss on ignition.

**Table 4:** The standard Gibbs energy change for reactions (1) to (4).

Chemical Reaction	ΔG°_T_ (kJ)		
	20 °C	80 °C	
$PbO+NaOH=Na^{+}+HPbO_{2}^{-}$	−32.569	−31.128	(1)
$ZnO+2NaOH+H_{2}O=2Na^{+}+Zn(OH{)}_{4}^{2-}$	−86.151	−78.120	(2)
$2HPbO_{2}^{-}+2Na^{+}+{3H}_{2}SO_{4}=Na_{2}{SO}_{4}+2PbSO_{4(s)}+4H_{2}O$	−407.395	−440.306	(3)
$2ZnO_{2}^{2-}+2Na^{+}+{2H}_{2}SO_{4}=Na_{2}{SO}_{4}+2ZnSO_{4(aq)}+2H_{2}O$	−231.576	−246.813	(4)

(aq)—“aqueous solution”, (s)—“solid”.

## Data Availability

The original contributions presented in the study are included in the article, further inquiries can be directed to the corresponding author.
